# Resveratrol inhibits lipid accumulation in the intestine of atherosclerotic mice and macrophages

**DOI:** 10.1111/jcmm.14323

**Published:** 2019-04-07

**Authors:** Guozhu Ye, Guoyou Chen, Han Gao, Yi Lin, Xu Liao, Han Zhang, Xinyu Liu, Yulang Chi, Qiansheng Huang, Huimin Zhu, Yuhua Fan, Sijun Dong

**Affiliations:** ^1^ Center for Excellence in Regional Atmospheric Environment, Institute of Urban Environment, Chinese Academy of Sciences Xiamen China; ^2^ Key Laboratory of Urban Environment and Health Institute of Urban Environment, Chinese Academy of Sciences Xiamen China; ^3^ College of Pharmacy Harbin Medical University‐Daqing Daqing Heilongjiang Province China; ^4^ University of Chinese Academy of Sciences Beijing China; ^5^ CAS Key Laboratory of Separation Science for Analytical Chemistry Dalian Institute of Chemical Physics, Chinese Academy of Sciences Dalian China

**Keywords:** atherosclerosis, cholesterol, fatty acid, intestinal, macrophage, peroxisome proliferator‐activated receptor, resveratrol

## Abstract

Disordered intestinal metabolism is highly correlated with atherosclerotic diseases. Resveratrol protects against atherosclerotic diseases. Accordingly, this study aims to discover novel intestinal proatherosclerotic metabolites and potential therapeutic targets related to the anti‐atherosclerotic effects of resveratrol. An untargeted metabolomics approach was employed to discover novel intestinal metabolic disturbances during atherosclerosis and resveratrol intervention. We found that multiple intestinal metabolic pathways were significantly disturbed during atherosclerosis and responsive to resveratrol intervention. Notably, resveratrol abolished intestinal fatty acid and monoglyceride accumulation in atherosclerotic mice. Meanwhile, oleate accumulation was one of the most prominent alterations in intestinal metabolism. Moreover, resveratrol attenuated oleate‐triggered accumulation of total cholesterol, esterified cholesterol and neutral lipids in mouse RAW 264.7 macrophages by activating ABC transporter A1/G1‐mediated cholesterol efflux through PPAR (peroxisome proliferator‐activated receptor) α/γ activation. Furthermore, we confirmed that PPARα and PPARγ activation by WY14643 and pioglitazone, respectively, alleviated oleate‐induced accumulation of total cholesterol, esterified cholesterol and neutral lipids by accelerating ABC transporter A1/G1‐mediated cholesterol efflux. This study provides the first evidence that resveratrol abolishes intestinal fatty acid and monoglyceride accumulation in atherosclerotic mice, and that resveratrol suppresses oleate‐induced accumulation of total cholesterol, esterified cholesterol and neutral lipids in macrophages by activating PPARα/γ signalling.

## 1 INTRODUCTION

Atherosclerosis (AS) is the leading cause of cardiovascular and cerebrovascular diseases, which are the most prevalent illnesses and causes of death worldwide.^1^, ^2^ Although the mortality rate for cardiovascular and cerebrovascular disease are declining globally, the absolute number of deaths is still growing.^1^ AS is a progressive metabolic disease characterized by lipid accumulation in the arteries and is highly correlated with metabolic abnormalities in lipids, carbohydrates, short chain fatty acids, amino acids and other metabolites.^1^, ^3^, ^4^, ^5^, ^6^, ^7^, ^8^, ^9^

Accumulating evidence has revealed that metabolic disturbances in the intestine are closely associated with atherosclerotic cardiovascular diseases.^4^, ^9^, ^10^ Intestinal microbial fermentation of carbohydrates generates short chain fatty acids, which increases energy availability and insulin sensitivity, promotes immune homeostasis and exhibit anti‐inflammatory effects.^9^, ^13^ In addition, secondary bile acids formed in the intestine can enhance insulin sensitivity, energy expenditure and brown adipose tissue activation and repress inflammation.^9^ Nevertheless, intestinal microbial conversion of phosphatidylcholine to trimethylamine accelerates cardiovascular disease.^14^, ^15^ Once formed from trimethylamine via hepatic flavin monooxygenases, trimethylamine *N*‐oxide accelerates cholesterol accumulation in macrophages by increasing forward cholesterol transport and decreasing reverse cholesterol transport.^4^, ^14^ Trimethylamine *N*‐oxide reduces cholesterol absorption by enterocytes, as well as bile acid synthesis and transport in the liver, and promotes lipopolysaccharide uptake by macrophages during AS.^4^, ^14^, ^15^

Notably, resveratrol (RSV), a natural plant polyphenol, can alleviate and even abrogate AS by intervening in various pathophysiological processes, including intestinal metabolism.^11^, ^16^, ^17^ RSV decreased intestinal microbial trimethylamine content, subsequently resulting in reduced liver trimethylamine *N*‐oxide synthesis and then preventing AS.^11^ Moreover, RSV promotes faecal bile acid excretion by activating bile salt hydrolase, leading to decreased ileal bile acid content and subsequent suppression of the enterohepatic farnesoid X receptor‐fibroblast growth factor 15 axis.^11^ The subsequent activation of liver cholesterol 7a‐hydroxylase triggers bile acid neosynthesis and facilitates cholesterol homeostasis, ultimately alleviating AS.^11^

Although much attention has been paid to the role of intestinal microbial metabolites in AS, we hypothesize that there are other intestinal metabolites that accelerate AS. Accordingly, to discover and verify novel proatherosclerotic metabolites and potential therapeutic targets related to the anti‐atherosclerotic effects of RSV, intestinal tissue was collected from mice in the control (ApoE^−/−^ mice fed a standard chow diet), AS (ApoE^−/−^ mice fed a high‐fat diet) and RSV intervention (ApoE^−/−^ mice fed a high‐fat diet and administered RSV) groups, and analysed using an untargeted metabolomics approach. Subsequently, the effects of proatherosclerotic metabolites and potential therapeutic targets were further confirmed in vitro.

## 2 MATERIALS AND METHODS

### 2.1 Chemicals and materials

Standard chow and high‐fat (standard chow with 0.15% cholesterol and 21% fat) diets were obtained from Nanjing Junke Biological Engineering Co. Ltd. (Nanjing, China). Methanol (HPLC grade) was purchased from Honeywell Burdick & Jackson (Muskegon, USA). Pyridine (anhydrous, 99.8%), *N*‐methyl‐*N*‐(trimethylsilyl)‐trifluoroacetamide (≥98.5%), methoxyamine hydrochloride (98%) and tridecanoic acid (≥98%) were from Sigma‐Aldrich (Shanghai, China). RAW264.7 macrophages were obtained from the Cell Bank of Chinese Academy of Science (Shanghai, China). Primers were all synthesized by Shanghai Sangon Biotech (Shanghai, China). DMEM (high glucose) was obtained from HyClone (USA). DMSO (≥99.7%), oleic acid (99.0%), RSV (99.0%), WY‐14643 and pioglitazone were purchased from Sigma‐Aldrich (Shanghai, China).

### 2.2 Mouse model and sample collection

All animal experiments in this study were approved by the Institutional Animal Ethics Committee of Harbin Medical University‐Daqing, performed in accordance with the guidelines of our Institutional Animal Care and Use Committee [Protocol (2009)‐11] and the UK Animals (Scientific Procedures) Act, 1986, and complied with ARRIVE guidelines.^18^ Male ApoE^−/−^ mice (8‐10 weeks, 22 ± 2 g) were purchased from Nanjing Junke Biological Engineering Co. Ltd. (Nanjing, China). Experimental modelling was initiated after good adaptive feeding for 1 week. According to our previous studies and other evidence, long‐term administration (8‐24 weeks) with RSV (5‐25 mg/kg) protects against AS.^19^, ^20^ Therefore, ApoE^−/−^ mice in the control and AS group were fed a standard chow diet and a high‐fat diet for 24 weeks, respectively. Meanwhile, ApoE^−/−^ mice in the RSV intervention group were fed a high‐fat diet and orally administered 10 mg/kg RSV twice daily for 24 weeks. After the 24‐week treatment, mice were randomly taken from each group, to evaluate aortic AS. The remaining mice were anaesthetized and sacrificed using pentobarbital sodium. Intestinal tissue and serum samples from each mouse were collected and placed in liquid nitrogen, followed by storage at −80°C for subsequent metabolomics analysis.

### 2.3 Cell culture and treatments

RAW264.7 cells were cultured in DMEM (high glucose, HyClone) supplemented with 10% foetal bovine serum at 37°C in a humidified 5% CO_2_ atmosphere. Macrophages were passaged every 3 days at a ratio of 1:4. After 12 passages, macrophages were treated with 65 μg/ml oleic acid, 65 μg/ml oleic acid plus RSV (1.5 μg/ml) or 1‰ DMSO as the control for 24 h, to explore the role of RSV in protecting against lipid accumulation. Furthermore, RAW264.7 cells were treated with 65 μg/ml oleic acid, 65 μg/ml oleic acid plus WY‐14643 (1.5 μg/ml), 65 μg/ml oleic acid plus pioglitazone (1.5 μg/ml) or 1‰ DMSO as the control for 24 h, to examine the role of PPARs in the protective effects of RSV on abrogating oleate‐induced lipid accumulation.

### 2.4 Nile red staining

RAW264.7 cells were fixed with 4% paraformaldehyde for 40 minutes and then washed with PBS three times. Nuclei were stained with DAPI (2.5 μg/ml, in methanol) for 15 minutes at 37°C followed by washing with methanol. Then, the cells were stained with Nile red (10 μg/ml, in methanol) for 0.5 hours at 37°C. After extensive washing with PBS, confocal fluorescence images were obtained using a confocal microscope (Zeiss, Germany). Cellular lipid content was measured by determining the integrated density ratio (Nile red/DAPI).

### 2.5 Tissue sample preparation for metabolomics investigation

After being snipped off and weighted accurately, frozen intestinal tissue was put in a centrifuge tube. A zirconia ball and ice‐cold 80% methanol were successively added, and the tissue was subjected to homogenization for 1.5 minutes at 25 Hz in a grinding and mixing apparatus (MM400, Retsch, Germany). After the mixture in the tube was centrifuged at 14 480 *g* for 15 minutes at 4°C, the supernatant was drawn and vacuum‐dried in a SpeedVac concentrator (Thermo Scientific, USA). Subsequently, the dried sample was dissolved in methoxyamine solution (20 mg/ml in pyridine) and vortexed for 30 seconds. Following an oximation reaction with methoxyamine in a 37°C water bath for 1.5 hours, the sample was put in a 37°C water bath for 1.0 hours for the silylation reaction with *N*‐methyl‐*N*‐(trimethylsilyl)‐trifluoroacetamide. Finally, the supernatant of the derivatized sample was collected for instrumental analysis after centrifugation. To monitor the stability and reproducibility of the metabolomics approach, quality control samples were prepared by mixing the remaining supernatant of all the analytical samples and treated in the same way as the analytical samples with regard to vacuum‐drying, derivatization, instrumental analysis and data processing.

### 2.6 Serum sample preparation for metabolomics investigation

After the serum sample was thawed at room temperature and vortexed for 10 seconds, 50 μL was removed and placed into a centrifuge tube on an ice bath. Two hundred microliters of methanol containing 15 μg/ml tridecanoic acid as the internal standard was added to the serum sample and vortexed for 30 seconds. Following centrifugation at 14 480 *g* for 15 minutes at 4°C, the supernatant of the serum sample was drawn and vacuum‐dried in a SpeedVac concentrator (Thermo Scientific, USA). Then, the dried sample was kept in a 37°C water bath for the 1.5 hours oximation reaction followed by the 1.0 hour silylation reaction. After centrifugation, the supernatant of the derivatized sample was collected for instrumental analysis.

### 2.7 Instrumental analysis for metabolomics investigation

For metabolic profiling, 1 μL of the derivatized sample was injected into a gas chromatography‐mass spectrometry systems via an AOC‐20i autosampler (GCMS‐QP 2010 plus, Shimadzu, Japan). The instrument parameters were set according to those used in our previous work.^22^, ^23^ Metabolites were separated on a DB‐5 MS capillary column (30 m × 250 μm × 0.25 μm, J&W Scientifc Inc, USA). Helium was utilized as the carrier gas, and the constant linear velocity and split ratio was set to 40.0 cm/s and 5:1, respectively. The oven temperature was initially set to 70°C for 3.0 minutes, then rose to 300°C at a rate of 5°C/min, and remained at 300°C for 10 min. The inlet, interface, and ion source temperatures were maintained at 300, 280 and 230°C, respectively. The ionization mode was electron impact (70 eV), and the detector voltage was set in accordance with the tuning result. Mass signals (m/z, 33‐600) were obtained in full scan mode. The event time and solvent delay time were 0.2 seconds and 5.3 minutes, respectively. Finally, a light diesel sample was analysed under the same conditions as the other samples to obtain the retention indices of metabolites.

### 2.8 Data preprocessing for metabolomics investigation

Peak matching and deconvolution were performed using XCMS and ChromaTOF 4.43 (LECO Corporation, USA), respectively.^25^ Metabolite identification was performed mainly based on the search results of commercial mass spectra libraries, and further verified by available reference standards according to the mass spectra, retention index and retention time. For metabolite quantification in the tissue sample, the raw peak area of metabolites were normalized to total ion current, and then multiplied by 1 × 10^7^. In addition, for metabolite quantification in the serum sample, the raw peak areas of metabolites were normalized to that of tridecanoic acid, and then multiplied by the concentration of tridecanoic acid.

### 2.9 RNA extraction and RT‐PCR analysis

Total RNA from RAW264.7 cells was extracted using TRIzol reagent (Thermo Fisher Scientific, MA, USA). RNA was reverse‐transcribed to cDNA with PrimeScript™ RT master mix (Takara, Dalian, China). Real‐time RT‐PCR was performed using SYBR^®^ Premix Ex Taq^™^ II (Takara, Dalian, China). β‐Actin was used as the internal standard for normalizing gene expression. The data were calculated using the 2^–ΔΔCt^ method. Q‐PCR primers were designed using the NCBI database (Table S1).

### 2.10 Determination of intracellular and extracellular total, free and esterified cholesterols

Intracellular and extracellular TC (total cholesterol) and FC (free cholesterol) levels were detected using a Cholesterol/Cholesteryl Ester Quantitation Assay kit (Abcam, USA). The results were normalized to the protein content according to the manufacturer's instructions. The CE (esterified cholesterol) content was determined by subtracting FC from TC.

### 2.11 Western blot analysis

The protein from RAW264.7 cell was obtained with cold cell lysis buffer and measured using the Bradford protein assay (Bio‐Rad, Hercules, CA, USA). 30 μg of protein was separated on 10% SDS‐PAGE gel and transferred to a nitrocellulose membrane. The membrane was blocked with 5% skimmed milk and then incubated with primary antibodies (Abcam, USA) at 4°C overnight. Subsequently, the membranes were incubated with appropriate secondary antibodies at room temperature for 1.5 hours. After the membranes were washed with TBST three times, protein bands were detected using a Tanon 6100 Chemiluminescent imaging system (Tanon, Shanghai, China).

### 2.12 Statistical analysis

MetaboAnalyst 3.0 was used for principal component analysis and pathway analysis.^26^ Two‐tailed Mann‐Whitney *U* tests and two‐independent‐sample *t* tests were performed using MeV 4.9.0 and PASW Statistics 18 software (SPSS Inc, Chicago, USA), respectively, to evaluate differences in metabolite levels, mRNA expression and fluorescence intensity between the groups.^27^ The heat map plot was generated using MeV 4.9.0. The level of significance was 0.05.

## 3 RESULTS

### 3.1 Significant intestinal metabolic changes during AS and RSV intervention

**Figure 1:**
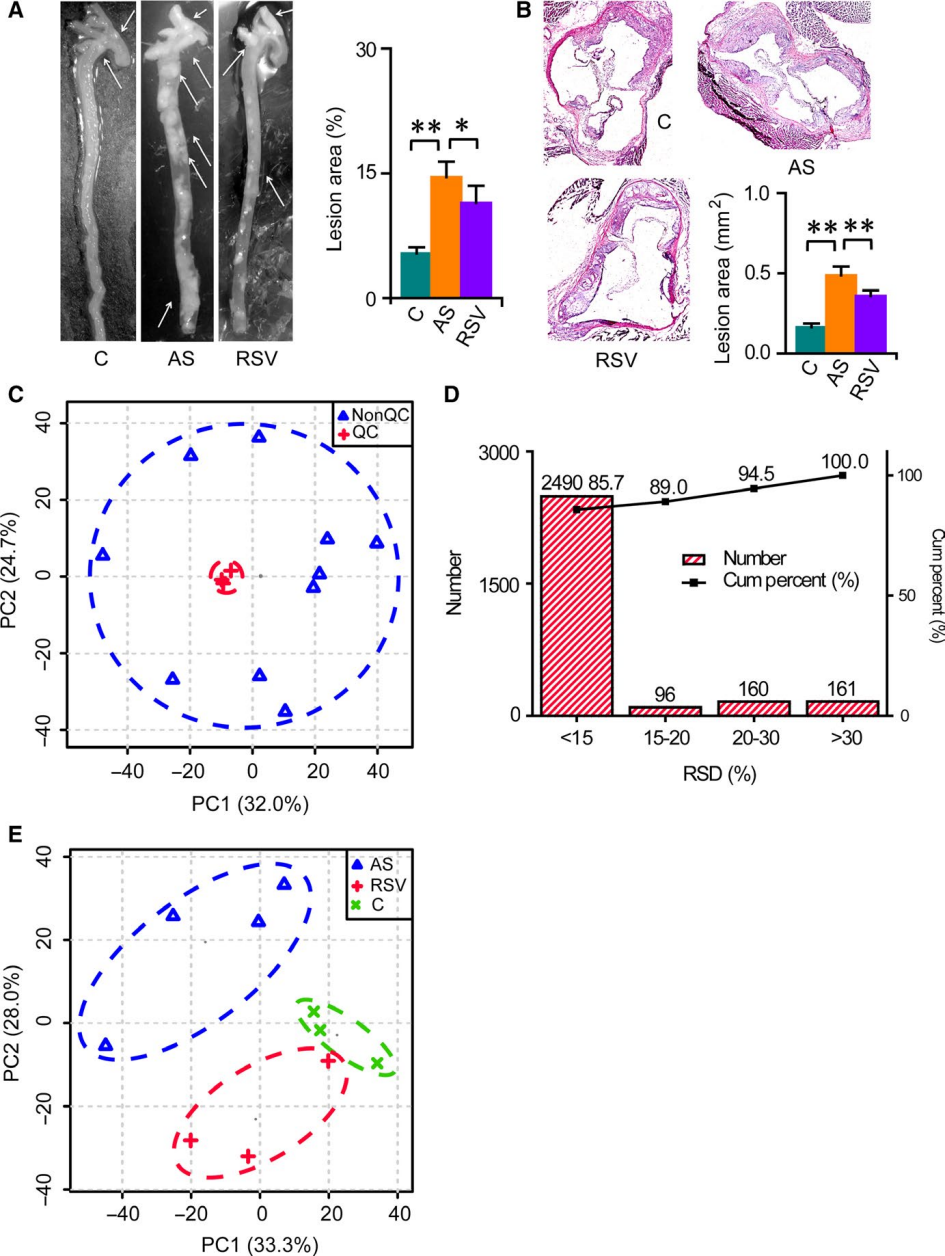
Substantial changes in the intestinal metabolic profile during AS and RSV intervention. C, AS and RSV denotes the control, atherosclerosis and RSV intervention group, respectively. Columns denote the mean + SD. *P < 0.05, **P < 0.01, two‐independent‐sample *t* test. A, Representative en face preparations of the total aorta in mice after 24‐week treatments and the quantitative result. n = 5 per group. B, Representative HE staining of aortic sinus in mice after 24‐week treatments and the quantitative result. n = 5‐6 per group. C, Analysis of the metabolic profiling. D, Distribution of the relative standard deviation (RSD) of the ions in the quality control (QC) sample. E, Changes in the intestinal metabolic profile during AS and RSV intervention

The prevalence of atherosclerotic lesions was evaluated in both the branchiocephalic arteries and aortic sinus from the mouse in each group after 24‐week treatments. As expected, atherosclerotic plaques in both the branchiocephalic arteries and aortic sinus were dominantly increased in ApoE^−/−^ mice in the AS group after 24‐week high‐fat diet treatment, while RSV treatment inhibited the progression of atherosclerosis (Figure 1A,B). Subsequently, an untargeted metabolomics approach based on gas chromatography‐mass spectrometry was employed to obtain intestinal metabolic signatures and identify key regulatory factors associated with AS and anti‐atherosclerotic effects of RSV. The three quality control samples were clustered together in the principle component analysis score plot, and the relative standard deviation of the levels in 85.7%, 89.0% and 94.5% of the 2907 ion peaks were below 15%, 20% and 30% in the quality control samples, respectively (Figure 1C,D). These data demonstrated the repeatability and stability of the metabolomics approach.^23^, ^24^, ^28^, ^29^

**Figure 2:**
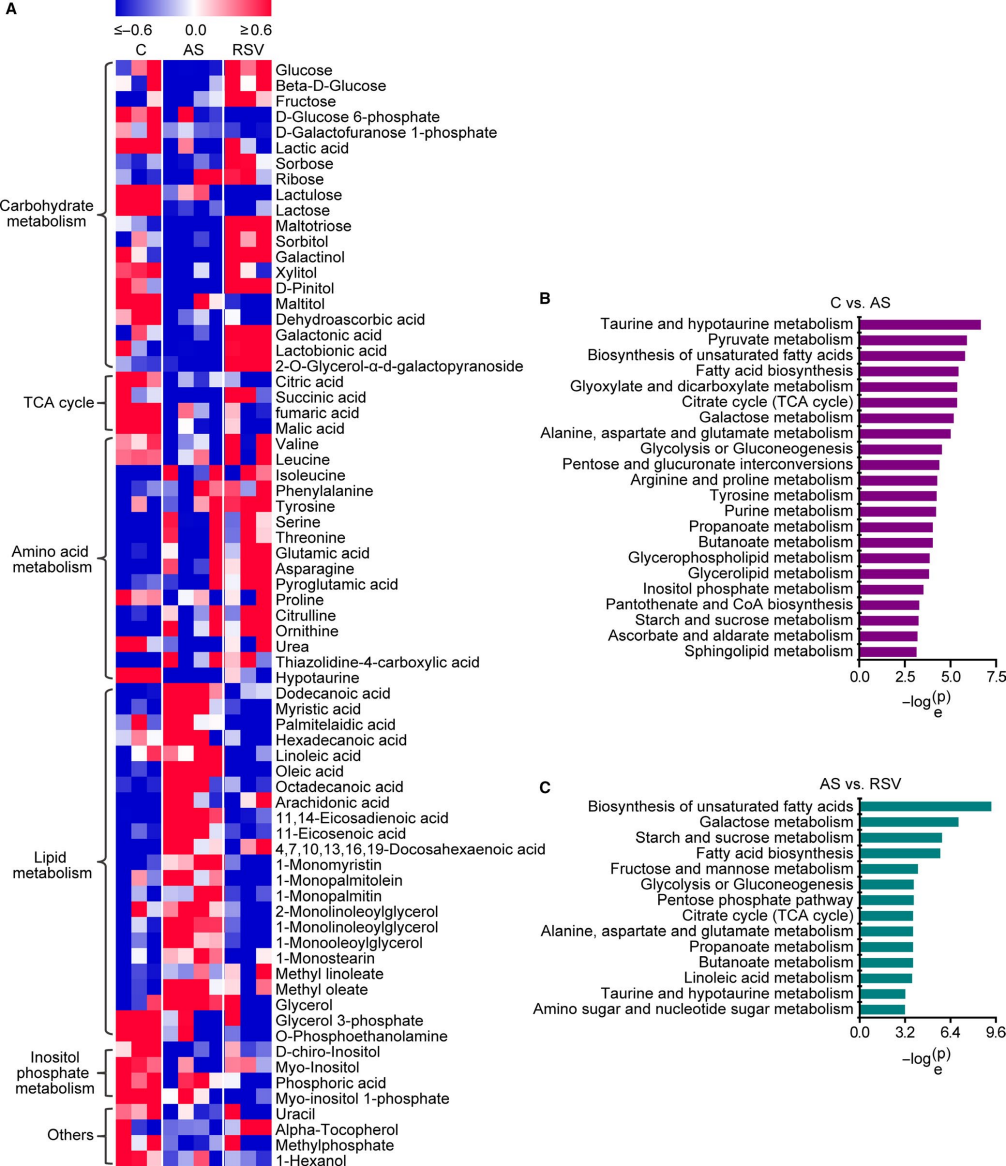
Substantial metabolic changes during AS and RSV intervention. n = 3, 4 and 3 in the control, AS and RSV group, respectively. A, Heat map of metabolic changes during AS and RSV intervention. Metabolite levels were normalized to the average and divided by the standard deviation. Then, the data were used to generate heat map. B, Pathway analysis of intestinal metabolic changes during AS. Significantly disturbed pathways are displayed (*P* < 0.05). C, Pathway analysis of intestinal metabolic changes during RSV intervention. Significantly disturbed pathways are displayed (*P* < 0.05)

The principal component analysis score plot showed that the intestinal metabolic profiling of the control group differed greatly from that of the AS group, and a significant difference was also observed in the metabolic profile between the AS and RSV groups (Figure 1E). Then, 71 of 168 detected metabolites were identified as differential metabolites related to AS and the effects of RSV. Among them, 57 metabolites were further confirmed by available standards (Table S2). The heat map showed substantial changes in intestinal metabolites involved in carbohydrate metabolism, tricarboxylic acid cycle, amino acid metabolism, lipid metabolism and other metabolic pathways during AS and RSV intervention (Figure 2A). Further pathway analysis revealed that 22 and 14 metabolic pathways were significantly disturbed during AS and RSV intervention, including biosynthesis of unsaturated fatty acids; fatty acid biosynthesis; and glycolysis/gluconeogenesis (Figure 2B,C). Detailed metabolic changes during AS and RSV intervention are described and discussed below.

### 3.2 RSV abolishes intestinal fatty acid and monoglyceride accumulation in atherosclerotic mice

**Figure 3:**
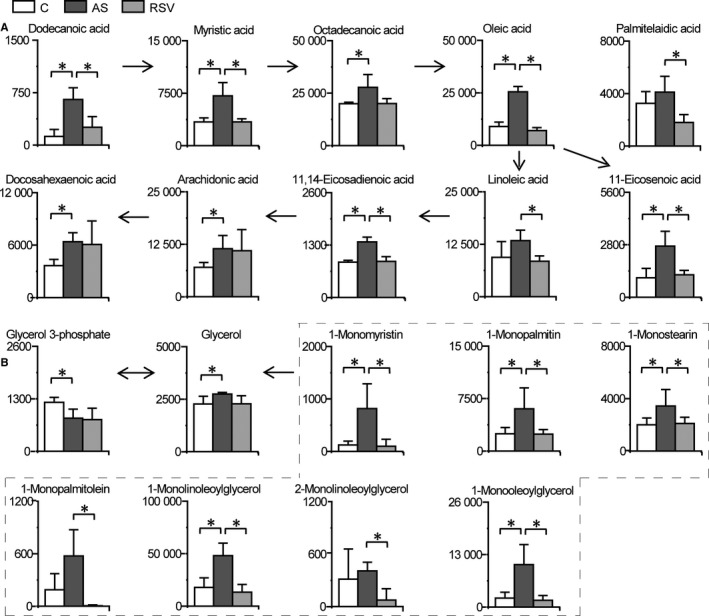
RSV abolishes intestinal fatty acid and monoglyceride accumulation in atherosclerotic mice. Columns denote the mean + SD. n = 3, 4 and 3 in the control, AS and RSV group, respectively. *P < 0.05, two‐tailed Mann‐Whitney *U* test. A, Changes in intestinal fatty acids during AS and RSV intervention. B, Changes in intestinal monoglycerides during AS and RSV intervention

Significant changes in lipid metabolism in the intestine were observed during AS and RSV intervention (Figures 2 and 3). Intestinal dodecanoic acid, myristic acid, oleate, 11‐eicosenoic acid and 11,14‐eicosadienoic acid levels were significantly higher in the AS group than in the control group, and significantly lower in the RSV group than in the AS group. Intestinal palmitelaidic acid and linoleic acid were significantly lower in the RSV group than in the AS group. The changes in fatty acids demonstrated that RSV effectively inhibited intestinal fatty acid accumulation in atherosclerotic mice. Similar changes in intestinal monoglycerides with fatty acids were observed during AS and RSV intervention. Intestinal 1‐monomyristin, 1‐monopalmitin, 1‐monostearin, 1‐monolinoleoylglycerol and 1‐monooleoylglycerol were significantly increased in the AS group compared with the levels in the control group, and significantly decreased in the RSV group compared with the levels in the AS group. Additionally, intestinal 1‐monopalmitolein and 2‐monolinoleoylglycerol were significantly reduced in the RSV intervention group compared with the levels in the AS group. These changes in monoglycerides illustrated that RSV effectively inhibited intestinal monoglyceride accumulation in atherosclerotic mice. Taken together, these data show that changes in fatty acids and monoglycerides in the intestine are closely correlated with AS and the anti‐atherosclerotic effects of RSV.

### 3.3 RSV attenuates changes in carbohydrate metabolism, amino acid metabolism and other pathways

**Figure 4:**
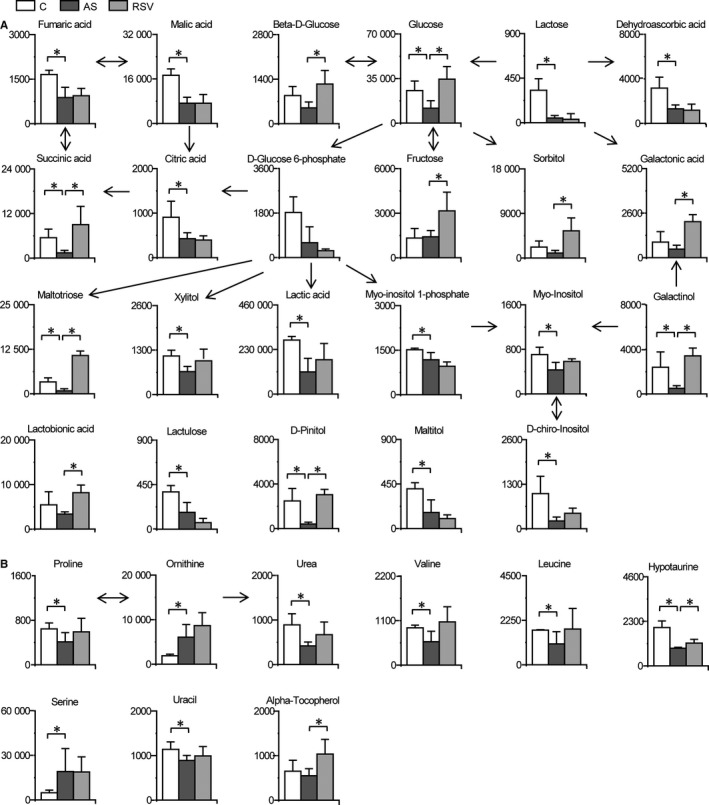
RSV attenuates changes in carbohydrate metabolism, amino acid metabolism and other pathways. Columns denote the mean + SD. n = 3, 4 and 3 in the control, AS and RSV group, respectively. *P < 0.05, two‐tailed Mann‐Whitney *U* test. A, Metabolic changes in intestinal carbohydrate metabolism during AS and RSV intervention. B, Metabolic changes in intestinal amino acid metabolism and other metabolic pathways during AS and RSV intervention

Significant changes in carbohydrate metabolism in the intestine also occurred during AS and RSV intervention (Figures 2 and 4). Most intestinal saccharides, such as glucose, beta‐D‐glucose, fructose, sorbitol, galactonic acid, galactinol and lactobionic acid, were significantly decreased during AS, and the decrease was alleviated or eliminated by RSV. In addition, intermediates in the tricarboxylic acid cycle (fumarate, malate, succinate and citrate) and lactic acid were also reduced in the AS group compared with the levels in the control group. Also the decreases in succinate and lactic acid during AS were alleviated or eliminated by RSV. The changes in carbohydrates indicate potential roles for intestinal carbohydrate metabolism (such as carbohydrate degradation into organic acids, inositol phosphate metabolism and glycolysis/gluconeogenesis) in AS and the anti‐atherosclerotic effects of RSV.

Disturbances in intestinal amino acid metabolism and other pathways occurred during AS and RSV intervention (Figures 2, 4 and Table S2). Intestinal proline, urea, valine, leucine and hypotaurine were significantly decreased in the AS group compared with the levels in the control group, and the decreases were alleviated to some extent by RSV. These changes in amino acid metabolism indicate the potential role of intestinal proline and arginine metabolism; valine, leucine and isoleucine metabolism; and taurine and hypotaurine metabolism in AS and the anti‐atherosclerotic effects of RSV.

### 3.4 RSV abolishes oleate‐induced lipid accumulation in macrophages

**Figure 5:**
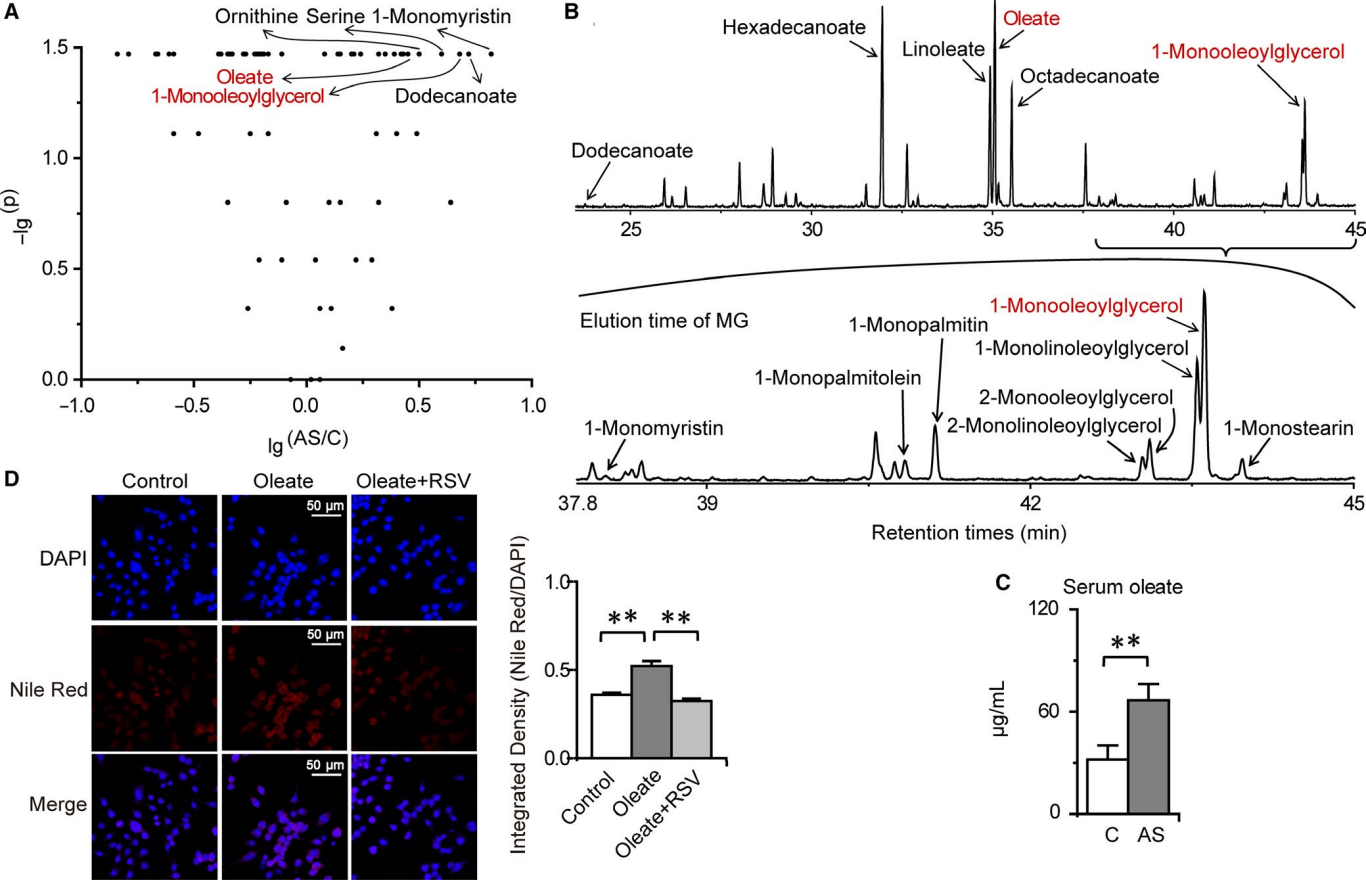
RSV abolishes oleate‐induced lipid accumulation in macrophages. Columns denote the mean + SD. **P < 0.01, two‐independent‐sample *t* test. A, Volcano plots of intestinal metabolite changes during AS. B, Typical total ion current chromatograms of intestinal fatty acids and monoglycerides. C, Changes in serum oleate during AS (n = 3 per group). D, Effects of oleate and RSV on lipid accumulation in macrophages (n = 6‐7 per group)

The volcano plot showed that 1‐monomyristin, dodecanoate, 1‐monooleoylglycerol, serine, orinithine and oleate were the differential metabolites with the largest fold changes in the comparison between the control and AS groups (Figure 5A). In addition, oleate and 1‐monooleoylglycerol were the predominant fatty acid and monoglyceride, respectively (Figure 5B). Moreover, intestinal fatty acid and monoglyceride accumulation was closely correlated with AS and the anti‐atherosclerotic effects of RSV. Therefore, we investigated the potential role of oleate in advancing AS. Since intestinal oleate can be released into the blood and then has the potential to promote AS, serum oleate was first measured. As expected, serum oleate was significantly increased in the AS group compared with that in the control group (Figure 5C). Given that lipid accumulation in macrophages is an important pathogenic event in AS, from fatty streak formation to atherosclerotic plaque rupture and myocardial infarction, the role of oleate in lipid accumulation in RAW 264.7 macrophages was further explored. Nile red staining demonstrated that neutral lipids were significantly enhanced in macrophages treated with 65 μg/ml oleate (the average serum concentration in atherosclerotic mice) compared with that in the control group, and the lipid accumulation in macrophages was completely abolished by RSV (Figure 5D). These data reveal that oleate induces lipid accumulation in macrophages and have the potential to advance AS. Moreover, RSV effectively abolished not only intestinal fatty acid accumulation in atherosclerotic mice, but also oleate‐induced lipid accumulation in macrophages.

### 3.5 RSV suppresses oleate‐induced TC and CE accumulation in macrophages by activating PPAR signalling

**Figure 6:**
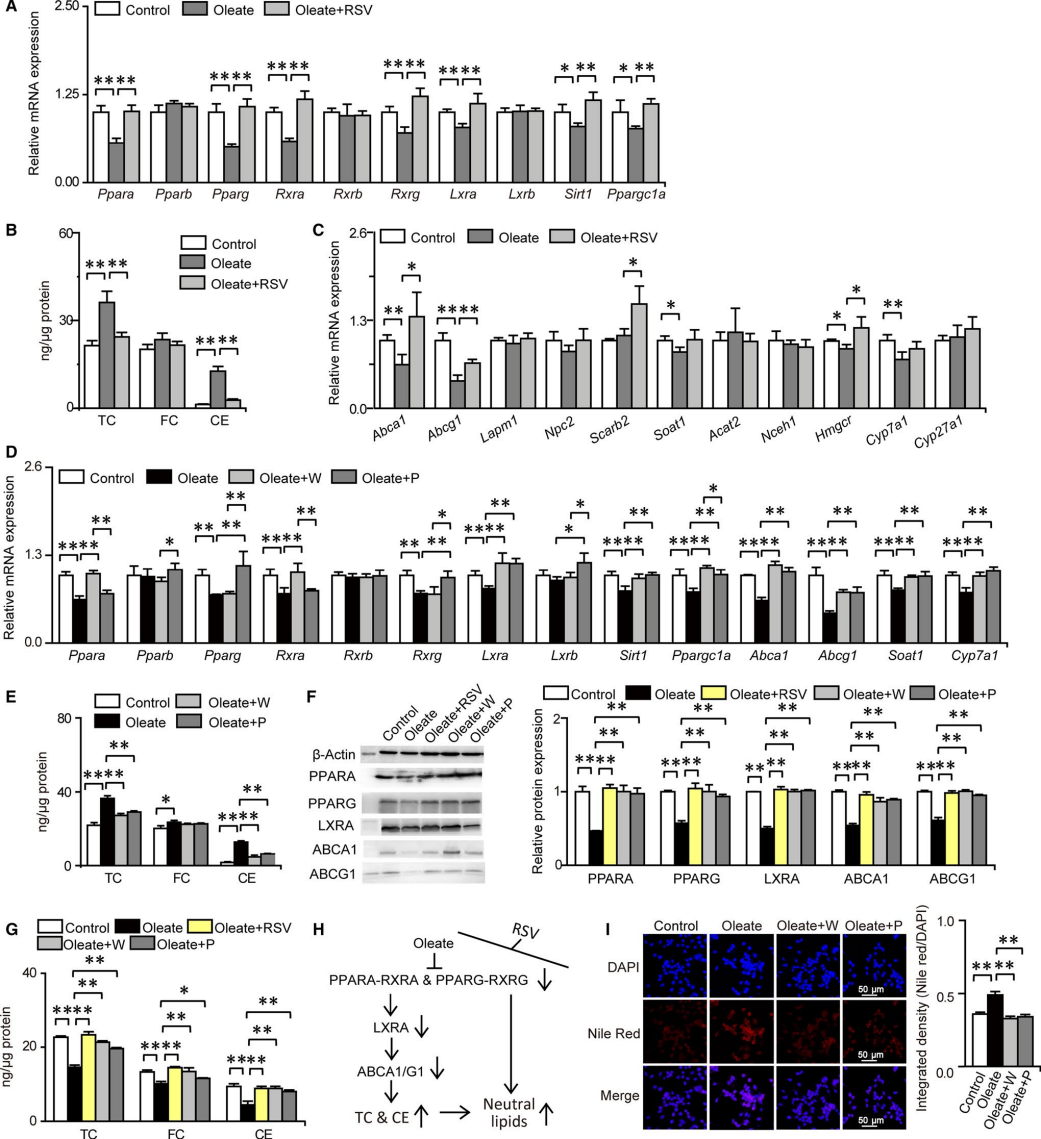
RSV abolishes oleate‐induced cholesterol and lipid accumulation in macrophages by activating PPAR signalling. Columns denote the mean + SD. *P < 0.05, **P < 0.01, two‐independent‐sample *t* test. A, RSV abolishes oleate‐induce changes in the mRNA expression of genes related to PPAR signalling (n = 4 per group). B, RSV inhibits oleate‐induced cholesterol accumulation in macrophages (n = 3 per group). C, RSV attenuates oleate‐induced changes in the mRNA expression of genes related to cholesterol transport, synthesis and catabolism (n = 4 per group). D, PPARα/γ activation abolishes oleate‐induced changes in the mRNA expression of genes related to cholesterol transport and metabolism (n = 4 per group). E, PPARα/γ activation attenuates oleate‐induced intracellular cholesterol accumulation (n = 3 per group). F, RSV abolishes oleate‐induced decreases in the expression levels of proteins related to cholesterol efflux. (n = 3 per group). G, RSV abolishes oleate‐induced decreases in extracellular cholesterol levels. (n = 3 per group). H, Schematic diagram of the molecular mechanisms whereby RSV abolishes oleate‐induced cholesterol and lipid accumulation in macrophages via PPARα/γ activation. Arrows on the right of molecular names: vertically upward‐/downward‐pointing solid arrows denote increases/decreases in response to oleate treatment, but the effects induced by oleate were abolished by RSV through activating PPARα/γ. I, PPARα/γ activation abolishes oleate‐induced lipid accumulation in macrophages (n = 6‐7 per group)

Fatty acids and their derivatives are natural ligands and/or mediators for PPARs, and PPARs acting as lipid sensors play crucial roles in lipid homeostasis.^30^, ^31^ To examine whether RSV abolishes lipid accumulation in macrophages treated with oleate via PPAR signalling, the mRNA expression of genes involved in PPAR signalling were measured. The mRNA expression levels of *Ppara*, *Pparg*, *Rxra*, *Rxrg*, *Lxra*, *Sirt1* and *Ppargc1a* were significantly reduced in RAW 264.7 macrophages treated with oleate compared with those in the control group, and these reductions were all completely eliminated by RSV (Figure 6A). However, changes in *Pparb* and *Rxrb* mRNA expression in response to the oleate treatment or RSV intervention were not significant (Figure 6A). These data suggest that RSV abolishes oleate‐triggered lipid accumulation by activating PPARα and PPARγ signalling. Given that PPARs have vital roles in cholesterol homeostasis and that cholesterol was the major lipid detected by Nile red staining and is the predominant lipid in the aorta, levels of TC, FC and CE were measured to determine the protective effects of RSV on cholesterol homeostasis.^32^, ^33^, ^36^, ^37^ We found that the significant up‐regulation of TC and CE in macrophages treated with oleate was largely abolished by RSV (Figure 6B). These results indicate that RSV inhibits oleate‐induced TC and CE accumulation in macrophages by stimulating PPARα and PPARγ signalling.

To understand how PPARα and PPARγ signalling influences the protective effects of RSV on reducing cholesterol accumulation in macrophages treated with oleate, the mRNA expression of genes related to cholesterol transport, esterification, anabolism and catabolism were determined. We found that the decrease in *Abca1*, *Abcg1* and *Hmgcr* mRNA expression in macrophages treated with oleate was largely abolished and even reversed by RSV, which indicated that the decrease in cholesterol efflux and de novo cholesterol synthesis induced by oleate was largely eliminated and even reversed after RSV intervention (Figure 6C). In addition, the *Scarb2* mRNA expression level was markedly enhanced in the RSV intervention group compared with that in the oleate treatment group, indicating that intracellular cholesterol transport to the cell membrane via the endosomal/lysosomal system was elevated in response to RSV intervention (Figure 6C). Thus, the protective effects of RSV on alleviating TC and CE accumulation in macrophages treated with oleate could be attributed to activated cholesterol efflux pathway through activation of PPAR signalling.

To further confirm the role of PPARα and PPARγ signalling in the protective effects of RSV on attenuating oleate‐induced cholesterol accumulation in macrophages, 0.1 μg/ml WY14643 and pioglitazone, selective agonists for PPARα and PPARγ, respectively, were separately added with oleate to the medium, and the mRNA expression of genes associated with PPAR signalling, cholesterol efflux pathway, and cholesterol esterification and catabolism were examined again. As anticipated, the significant decrease in *Ppara* and *Rxra* mRNA expression in macrophages treated with oleate was completely abolished by WY14643 but not by pioglitazone (Figure 6D). Meanwhile, the significant reduction in *Pparg* and *Rxrg* in macrophages induced by oleate was completely abolished by pioglitazone but not by WY14643 (Figure 6D). Moreover, the significant decrease in *Lxra*, *Sirt1*, *Ppargc1a*, *Abca1*, *Abcg1*, *Soat1* and *Cyp7a1* mRNA expression in macrophages induced by oleate was completely eliminated by both WY14643 and pioglitazone (Figure 6D). These data demonstrated the high specificity of PPARα and PPARγ signalling activated by WY14643 and pioglitazone, respectively, and the regulatory roles of PPARα and PPARγ signalling. Then, we further verified that the significant TC and CE accumulation in macrophages treated with oleate was largely alleviated or abolished by both WY14643 and pioglitazone (Figure 6E). Levels of proteins related to cholesterol efflux and contents of extracellular cholesterol were further measured to confirm the role of RSV in reducing cholesterol accumulation via PPAR‐mediated cholesterol efflux. The data showed that oleate‐induced decreased levels of PPARA/G, LXRA and ABCA1/G1 and reduced contents of extracellular TC, FC and CE were largely or completely abolished by both RSV and PPARα/γ activation, demonstrating the role of PPARα/γ‐mediated cholesterol efflux in the protective effects of RSV on eliminating cholesterol accumulation (Figure 6F,G). In summary, the protective effects of RSV on reducing TC and CE accumulation in macrophages treated with oleate was mainly due to activated cholesterol efflux pathway, which was mediated by PPARα and PPARγ signalling (Figure 6H). Finally, we also observed that the marked accumulation of neutral lipids in macrophages treated with oleate was completely eliminated by both WY14643 and pioglitazone, demonstrating that neutral lipid accumulation triggered by oleate was also controlled by PPARα and PPARγ (Figure 6I).

## 4 DISCUSSION

In this study, an untargeted metabolomics approach was employed to discover intestinal metabolic perturbation and potential therapeutic targets related to AS and the anti‐atherosclerotic effects of RSV. We found that many metabolites involved in carbohydrate metabolism, amino acid metabolism and lipid metabolism were significantly altered during AS and RSV intervention.

Most monosaccharides, disaccharides, trisaccharides and organic acids involved in the tricarboxylic acid cycle, glycolysis and organic acid metabolism were decreased during AS. However, the decreases in most saccharides, succinate and lactate were partially or completely abolished by RSV. These data suggest that intestinal absorption and/or metabolism of saccharides and organic acids were disturbed, and that short chain organic acid production from carbohydrate fermentation via lactate and/or succinate was inhibited during AS.^13^ In contrast, RSV promoted carbohydrate fermentation into short chain organic acids, which stimulated G‐protein‐coupled receptors, thereby enhancing intestinal insulin sensitivity, gluconeogenesis, transit rate and subsequent cholesterol homeostasis by facilitating faecal bile acid excretion.^40^, ^41^

Here, we found that the decreases in proline, urea, valine, leucine, hypotaurine and uracil during AS were attenuated by RSV, which demonstrated protective effects of RSV on alleviating disturbances in arginine and proline metabolism, taurine and hypotaurine metabolism, and branched‐chain amino acid metabolism. Disturbed arginine and proline metabolism led to intestinal absorption and secretion disorders and impaired endothelial functions in atherosclerotic mice.^42^, ^43^ In addition, the decreases in valine and leucine suggested decreased anaplerotic substrates from branched‐chain amino acids for replenishing tricarboxylic acid cycle intermediates through succinyl‐CoA or α‐ketoglutarate in atherosclerotic mice, which was demonstrated by the increases in valine, leucine and succinate induced by RSV. Disturbances in branched‐chain amino acids can trigger insulin resistance in skeletal muscle and predict cardiovascular disease.^45^ Moreover, the decline in hypotaurine indicated enhanced intestinal oxidative stress in atherosclerotic mice, which was consistent with the decreased dehydroascorbate in this study.^46^ Notably, iron‐overloaded mice treated with taurine, derived from hypotaurine, are protected from oxidative stress and myocardial lipid peroxidation.^47^

Notably, we also discovered that RSV abrogated intestinal fatty acid and monoglyceride accumulation in atherosclerotic mice. Following hydrolysis of dietary lipids, fatty acids and monoglycerides (the major hydrolysed products in the intestinal lumen) enter enterocytes through various transporters and are packed into chylomicrons in the form of triglycerides.^48^, ^49^ Subsequently, fatty acids and monoglycerides are delivered to the blood after chylomicron secretion by enterocytes and relevant lipoprotein hydrolysis.^48^, ^49^ Accordingly, protective effects of RSV on reducing intestinal fatty acid (including oleate) and monoglyceride accumulation were likely attributed to improved intestinal functions, such as reduced lipid absorption and secretion. Nonetheless, other metabolic abnormalities, such as adipocyte lipolysis, cannot be neglected. On the other hand, blood oleate in turn accelerates intestinal lipoprotein production and chylomicron secretion.^50^ Furthermore, increased intestinal lipid absorption due to increased chylomicron assembly and secretion contributes to hyperlipidaemia and formation of atherosclerotic plaques in ApoE^−/−^mice.^51^

Accordingly, protective effects of RSV on inhibiting oeate‐induced lipid accumulation in macrophages was further examined. We found that RSV abolished neutral lipid accumulation in macrophages treated with oleate at its average serum concentration in atherosclerotic mice by activating PPARα or PPARγ signalling. Moreover, oleate‐triggered accumulation of TC and CE was largely eliminated by RSV via promoting PPARα/γ‐mediated cholesterol efflux pathway. CE derived from either the intestine or liver has been found to significantly accelerate atherosclerotic progression.^52^

Oleate is a natural ligand for PPARs and influences the transcriptional control by PPARs, which requires heterodimerization with retinoid X receptor (RXR) and interaction with a coregulator complex.^30^ When binding to PPARα or PPARγ, oleate triggers conformational changes in the ligand‐binding domain and suppresses the expression of *Ppara* or *Pparg* and *Rxra* or *Rxrg*, leading to reduced formation of the PPARα‐RXRα or PPARγ‐RXRγ transcriptional complex. Oleate also induced decreased *Sirt1* and *Ppargc1a* expression and subsequently reduced recruitment of peroxisome proliferator‐activated receptor gamma coactivator 1‐α by PPARα or PPARγ, further suppressing the transcriptional regulation by PPARα‐RXRα or PPARγ‐RXRγ complex. Subsequently, the mRNA and protein expression levels of *Lxra*, *Abca1* and *Abcg1*, target genes of PPARα and PPARγ, were decreased, leading to reduced cholesterol efflux and resultant TC and CE accumulation in macrophages treated with oleate. Notably, above molecular events were alleviated or abolished by RSV via its activation of PPARα and PPARγ signalling.

To our knowledge, this is the first report that RSV abolishes intestinal fatty acid and monoglyceride accumulation in atherosclerotic mice, and that RSV suppresses oleate‐induced accumulation of TC, CE and neutral lipids in macrophages through the activation of PPAR signalling. This study suggests that replacing high‐oleate diets with low‐oleate diets, blocking intestinal absorption and secretion of fatty acids and monoglycerides, promoting cholesterol efflux, activating PPARα or PPARγ signalling, and RSV intervention would be beneficial for cardiovascular health and treatments.

## CONFLICT OF INTEREST

The authors declare that they have no conflicts of interest.

## AUTHOR CONTRIBUTION

GY, GC, YF and SD conceived and designed this study. GY performed the tissue and serum sample preparation, data processing for metabolomics analysis and subsequent statistical analysis, wrote and revised the manuscript. GC and YF carried out the animal experiment and sample collection. HG performed the cell experiment and Nile red staining, and detected levels of total FFAs, FC, TC, CE,mRNA and protein expression. YL, YC, QH and HZ participated in the tissue and serum preparation for metabolomics analysis. XL and HZ initiated the instrumental analysis. XYL deconvoluted the mass data. All authors have read and given approval of the final manuscript.
