# Supplementary material for: Resveratrol inhibits lipid accumulation in the intestine of atherosclerotic mice and macrophages
Source: J Cell Mol Med. 2019 Apr 7;23(6):4313–25. doi: 10.1111/jcmm.14323 (PMC6533483; doi:10.1111/jcmm.14323)
Supplement: Supplementary file 1 [file JCMM-23-4313-s001.docx]

**Table S1** Primers for RT-PCR detection.

| **Genes** | **Primer Sequence** |
| --- | --- |
| *Abca1* | F: TCGGCAGATACATTGAAAACCC  R: TCCTCCTGAATCTCAGACTTCCT |
| *Abcg1* | F: CCTTCCTCAGCATCATGCG  R: CCGATCCCAATGTGCGA |
| *Lamp1* | F: GCCTCAGCACTCTTTGAGGT  R: GCAGTCTCGTAGGTGGTCAG |
| *Npc2* | F: GAATGTGAGCCCATGTCCCA  R: GACTGAGTGCCGCTGGTAAA |
| *Scarb2* | F: CCTGCTCAGGGAGCTTATCG  R: GTTCGTGCACGGTGTGAATC |
| *Soat1* | F: CCGAGACAACTACCCAAGGA  R: CACACACAGGACCAGGACAC |
| *Acat2* | F: ATTGTTGAAAGGTGGGCAGC  R: GGTAACATCCCATCCCGTCA |
| *Nceh1* | F: GCCTACTACGTGTACATCCCA  R: TTCAGCGCGATCAGATGGT |
| *Hmgcr* | F: CAGGATGCAGCACAGAATGT  R: CTTTGCATGCTCCTTGAACA |
| *Cyp27a1* | F: TGGACAACCTCCTTTGGGAC  R: TTGCCCTCCTGTCTCATCAC |
| *Cyp7a1* | F: CACATACCTTCCCATGGCTT  R: CTTAGGCTGCACAGAAACGG |
| *Sirt1* | F: GAGCTGGGGTTTCTGTCTCC  R: CCGCAAGGCGAGCATAGATA |
| *Pgc1α* | F: GTGTTCTGGTACCCAAGGCA  R: ATGGTCACCAAACAGCCGAA |
| *Lxra* | F: CTGAAGCGGCAAGAAGAGGA  R: CTGTGGCAGGACTTGAGGAG |
| *Lxrb* | F: GCGGACACAGAGGCAACTC  R: CTCATGACTGCACCGGCGAT |
| *Ppara* | F: CTTCCCAAAGCTCCTTCAAAAA  R: CTGCGCATGCTCCGTG |
| *Pparb* | F: GATGACAGTGACCTGGCGCT  R: AGGCCTGGCCGGTCTC |
| *Pparg* | F: GACAGGAAAGACAACGGACAAA  R: GCTTCTACGGATCGAAACTGG |
| *Rxra* | F: TGTGGATCTTTGGGGTGCAG  R: TGAGTAAAGATGGCGAGAGTGG |
| *Rxrb* | F: CCTGACCTACTCGTGTCGTG  R: GCGACAGTACTGACAGCGAT |
| *Rxrg* | F: GAATGAACTGAGCAGCCCAAC  R: CAAGGCTACTGAAGGGCTCA |
| **Genes** | **Primer Sequence** |
| *β-Actin* | F: CTGGGACGACATGGAGAAGA  R: ACCAGAGGCATACAGGGACA |

**Table S2** Differential metabolites discovered from the comparisons among the animal groups.

| **Metabolites** | **Related pathways** | **AS vs. C** | | **AS vs. RSV** | | **RSV vs. C** | |
| --- | --- | --- | --- | --- | --- | --- | --- |
|  |  | **AS/C** | ***P*** | **RSV/AS** | ***P*** | **RSV/C** | ***P*** |
| Carbohydrate metabolism |  |  |  |  |  |  |  |
| Glucose^a^ | Glycolysis, gluconeogenesis, pentose phosphate pathway | **0.46** | **0.034** | **2.95** | **0.034** | 1.35 | 0.275 |
| Beta-D-Glucose^a^ | Glycolysis, gluconeogenesis, pentose phosphate pathway | 0.56 | 0.077 | **2.50** | **0.034** | 1.41 | 0.275 |
| Fructose^a^ | Fructose and mannose metabolism | 1.06 | 1.000 | **2.25** | **0.034** | **2.38** | **0.0495** |
| D-Glucose 6-phosphate^a^ | Glycolysis, gluconeogenesis, pentose phosphate pathway | 0.33 | 0.077 | 0.46 | 0.289 | **0.15** | **0.0495** |
| D-Galactofuranose 1-phosphate^b^ | Carbohydrate metabolism | 0.26 | 0.077 | **0.35** | **0.034** | **0.09** | **0.0495** |
| Lactic acid^a^ | Glycolysis | **0.41** | **0.034** | 1.55 | 0.289 | 0.64 | 0.127 |
| Sorbose^a^ | Fructose and mannose metabolism | 0.82 | 0.157 | **3.04** | **0.034** | **2.50** | **0.0495** |
| Ribose^a^ | Pentose phosphate pathway | 1.45 | 0.724 | 1.21 | 0.724 | **1.76** | **0.0495** |
| Lactulose^b^ | Carbohydrate metabolism | **0.45** | **0.034** | 0.39 | 0.157 | **0.17** | **0.0495** |
| Lactose^a^ | Galactose metabolism | **0.14** | **0.034** | 0.71 | 0.476 | **0.10** | **0.0463** |
| Maltotriose^a^ | Carbohydrate digestion and absorption | **0.25** | **0.034** | **13.05** | **0.034** | **3.24** | **0.0495** |
| Sorbitol^a^ | Fructose and mannose metabolism | 0.45 | 0.157 | **5.57** | **0.034** | 2.48 | 0.127 |
| Galactinol^a^ | Galactose metabolism | **0.21** | **0.034** | **6.72** | **0.034** | 1.43 | 0.275 |
| Xylitol^a^ | Pentose and glucuronate interconversions | **0.59** | **0.034** | 1.47 | 0.077 | 0.87 | 0.275 |
| D-Pinitol^b^ | Carbohydrate metabolism | **0.16** | **0.034** | **7.51** | **0.034** | 1.23 | 0.513 |
| Maltitol^a^ | Carbohydrate metabolism | **0.40** | **0.034** | 0.62 | 0.724 | **0.25** | **0.0495** |
| Dehydroascorbic acid^a^ | Ascorbate and aldarate metabolism, glutathione metabolism | **0.41** | **0.034** | 0.90 | 0.480 | **0.37** | **0.0495** |
| Galactonic acid^a^ | Galactose metabolism, pentose and glucuronate interconversions | 0.55 | 0.480 | **4.24** | **0.034** | **2.32** | **0.0495** |
| Lactobionic acid^a^ | Carbohydrate metabolism | 0.62 | 0.289 | **2.42** | **0.034** | 1.50 | 0.275 |
| 2-O-Glycerol-α-d-galactopyranoside^b^ | Carbohydrate metabolism | **0.53** | **0.034** | **6.17** | **0.034** | **3.25** | **0.0495** |
| Tricarboxylic acid (TCA) cycle |  |  |  |  |  |  |  |
| Citric acid^a^ | TCA cycle | 0.47 | 0.034 | 0.93 | 0.480 | 0.44 | 0.0495 |
| **Metabolites** | **Related pathways** | **AS vs. C** | | **AS vs. RSV** | | **RSV vs. C** | |
|  |  | **AS/C** | **P** | **RSV/AS** | **P** | **RSV/C** | **P** |
| Succinic acid^a^ | TCA cycle | **0.25** | **0.034** | **6.45** | **0.034** | 1.64 | 0.513 |
| Fumaric acid^a^ | TCA cycle | **0.53** | **0.034** | 1.07 | 0.724 | **0.57** | **0.0495** |
| Malic acid^a^ | TCA cycle | **0.42** | **0.034** | 1.00 | 0.480 | **0.42** | **0.0495** |
| Amino acid metabolism | | | | | | | |
| Valine^a^ | Valine, leucine and isoleucine metabolism | **0.63** | **0.034** | 1.85 | 0.157 | 1.16 | 0.513 |
| Leucine^a^ | Valine, leucine and isoleucine metabolism | **0.60** | **0.034** | 1.72 | 0.157 | 1.03 | 0.513 |
| Isoleucine^a^ | Valine, leucine and isoleucine metabolism | 1.96 | 0.289 | 1.08 | 0.724 | **2.12** | **0.0495** |
| Phenylalanine^a^ | Phenylalanine metabolism | 1.65 | 0.289 | 1.42 | 0.289 | **2.35** | **0.0495** |
| Tyrosine^a^ | Tyrosine metabolism | 1.16 | 0.480 | 1.17 | 0.157 | **1.35** | **0.0495** |
| Serine^a^ | Glycine, serine and threonine metabolism | **4.01** | **0.034** | 0.99 | 0.724 | **3.95** | **0.0495** |
| Threonine^a^ | Glycine, serine and threonine metabolism | 2.38 | 0.480 | 1.08 | 0.724 | **2.57** | **0.0495** |
| Glutamic acid^a^ | Alanine, aspartate and glutamate metabolism | 2.08 | 0.157 | 1.35 | 0.480 | **2.80** | **0.0495** |
| Asparagine^b^ | Alanine, aspartate and glutamate metabolism | 2.53 | 0.077 | 1.49 | 0.289 | **3.77** | **0.0495** |
| Pyroglutamic acid^a^ | Glutathione metabolism | 1.14 | 1.000 | 1.22 | 0.289 | **1.39** | **0.0495** |
| Proline^a^ | Arginine and proline metabolism | **0.64** | **0.034** | 1.43 | 0.289 | 0.92 | 0.513 |
| Citrulline^a^ | Arginine and proline metabolism | 2.04 | 0.077 | 1.15 | 0.724 | **2.34** | **0.0495** |
| Ornithine^a^ | Arginine and proline metabolism | **3.19** | **0.034** | 1.43 | 0.157 | **4.56** | **0.0495** |
| Urea^a^ | Arginine and proline metabolism | **0.47** | **0.034** | 1.60 | 0.157 | 0.75 | 0.275 |
| Thiazolidine-4-carboxylic acid^b^ | Amino Acid Metabolism | 4.38 | 0.157 | 1.05 | 1.000 | **4.62** | **0.0495** |
| Hypotaurine^a^ | Taurine and hypotaurine metabolism | **0.46** | **0.034** | **1.29** | **0.034** | **0.59** | **0.0495** |
| Lipid metabolism | | | | | | | |
| Dodecanoic acid^a^ | Fatty acid biosynthesis | **5.27** | **0.034** | **0.39** | **0.034** | 2.05 | 0.275 |
| Myristic acid^a^ | Fatty acid biosynthesis | **2.10** | **0.034** | **0.48** | **0.034** | 1.00 | 0.827 |
| Palmitelaidic acid^b^ | Fatty acid biosynthesis | 1.26 | 0.157 | **0.44** | **0.034** | **0.55** | **0.0495** |
| **Metabolites** | **Related pathways** | **AS vs. C** | | **AS vs. RSV** | | **RSV vs. C** | |
|  |  | **AS/C** | ***P*** | **RSV/AS** | ***P*** | **RSV/C** | ***P*** |
| Hexadecanoic acid^a^ | Fatty acid biosynthesis | 1.11 | 0.289 | 0.73 | 0.157 | **0.81** | **0.0495** |
| Linoleic acid^a^ | Fatty acid biosynthesis | 1.42 | 0.157 | **0.63** | **0.034** | 0.90 | 0.513 |
| Oleic acid^a^ | Fatty acid biosynthesis | **2.84** | **0.034** | **0.27** | **0.034** | 0.78 | 0.127 |
| Octadecanoic acid^a^ | Fatty acid biosynthesis | **1.39** | **0.034** | 0.72 | 0.077 | 1.00 | 0.513 |
| Arachidonic acid^a^ | Fatty acid biosynthesis | **1.63** | **0.034** | 0.96 | 1.000 | 1.57 | 0.275 |
| 11,14-Eicosadienoic acid^a^ | Fatty acid biosynthesis | **1.57** | **0.034** | **0.65** | **0.034** | 1.02 | 0.513 |
| 11-Eicosenoic acid^a^ | Fatty acid biosynthesis | **2.64** | **0.034** | **0.44** | **0.034** | 1.16 | 0.827 |
| 4,7,10,13,16,19-Docosahexaenoic acid^a^ | Fatty acid biosynthesis | **1.75** | **0.034** | 0.95 | 1.000 | 1.66 | 0.275 |
| 1-Monomyristin^b^ | Glycerolipid metabolism | **6.61** | **0.034** | **0.12** | **0.034** | 0.79 | 0.513 |
| 1-Monopalmitolein^b^ | Glycerolipid metabolism | 3.06 | 0.077 | **0.01** | **0.032** | 0.03 | 0.246 |
| 1-Monopalmitin^a^ | Glycerolipid metabolism | **2.45** | **0.034** | **0.40** | **0.034** | 0.98 | 0.827 |
| 2-Monolinoleoylglycerol^b^ | Glycerolipid metabolism | 1.30 | 0.480 | **0.18** | **0.032** | 0.24 | 0.246 |
| 1-Monolinoleoylglycerol^b^ | Glycerolipid metabolism | **2.70** | **0.034** | **0.28** | **0.034** | 0.75 | 0.827 |
| 1-Monooleoylglycerol^b^ | Glycerolipid metabolism | **4.74** | **0.034** | **0.16** | **0.034** | 0.75 | 0.827 |
| 1-Monostearin^a^ | Glycerolipid metabolism | **1.72** | **0.034** | **0.61** | **0.034** | 1.05 | 0.275 |
| Methyl linoleate^a^ | Lipid metabolism | **1.42** | **0.034** | 1.45 | 0.724 | 2.05 | 0.275 |
| Methyl oleate^b^ | Lipid metabolism | **2.24** | **0.034** | 0.64 | 0.157 | 1.43 | 0.275 |
| Glycerol^a^ | Glycerolipid metabolism, glucose synthesis, glycolysis | **1.21** | **0.034** | 0.83 | 0.077 | 1.00 | 0.827 |
| Glycerol 3-phosphate^a^ | Glycerolipid metabolism, glycolysis | **0.67** | **0.034** | 0.96 | 1.000 | **0.65** | **0.0495** |
| O-Phosphoethanolamine^a^ | Glycerophospholipid metabolism, sphingolipid metabolism | **0.54** | **0.034** | 0.68 | 0.157 | **0.37** | **0.0495** |
| Inositol phosphate metabolism | | | | | | | |
| D-*chiro*-Inositol^a^ | Inositol phosphate metabolism | **0.22** | **0.034** | 1.99 | 0.077 | 0.44 | 0.127 |
| Myo-Inositol^a^ | Inositol phosphate metabolism | **0.61** | **0.034** | 1.35 | 0.077 | **0.82** | **0.0495** |
| **Metabolites** | **Related pathways** | **AS vs. C** | | **AS vs. RSV** | | **RSV vs. C** | |
|  |  | **AS/C** | ***P*** | **RSV/AS** | ***P*** | **RSV/C** | ***P*** |
| Phosphoric acid^a^ | Inositol phosphate metabolism | 0.78 | 0.289 | 0.70 | 0.157 | **0.55** | **0.0495** |
| Myo-inositol 1-phosphate^b^ | Inositol phosphate metabolism | **0.78** | **0.034** | 0.81 | 0.157 | **0.63** | **0.0495** |
| Others | | | | | | | |
| Uracil^a^ | Pyrimidine metabolism | **0.78** | **0.034** | 1.11 | 1.000 | 0.87 | 0.275 |
| Alpha-Tocopherol^a^ | Ubiquinone and other terpenoid-quinone biosynthesis | 0.85 | 1.000 | **1.88** | **0.034** | 1.59 | 0.127 |
| Methylphosphate^a^ | Phosphonate and phosphinate metabolism | **0.61** | **0.034** | 1.05 | 0.724 | 0.64 | 0.127 |
| 1-Hexanol^b^ | ——— | 0.67 | 0.077 | 1.06 | 1.000 | **0.71** | **0.0495** |

^a^ Identified based on the search results of commercial mass spectra libraries, and further verified by available reference standards according to the mass spectra, retention time and retention index. ^b^ Identified based on the search results of commercial mass spectra libraries. A two-tailed Mann-Whitney U test was used to discover the differential metabolites (*P*<0.05). N=3, 4 and 3 in the control, AS and RSV intervention group, respectively. Red/blue bold fonts: significantly increased/decreased in the comparison (*P*<0.05).
